# A common root for coevolution and substitution rate variability in protein sequence evolution

**DOI:** 10.1038/s41598-019-53958-w

**Published:** 2019-12-02

**Authors:** Francesca Rizzato, Stefano Zamuner, Andrea Pagnani, Alessandro Laio

**Affiliations:** 1 Scuola Internazionale Superiore di Studi Avanzati (SISSA), Trieste, Italy; 2 DISAT, Politecnico di Torino, Torino, Italy; 3 Italian Institute for Genomic Medicine (IIGM), Torino, Italy; 4 Istituto Nazionale di Fisica Nucleare (INFN) Sezione di Torino, Torino, Italy; 5 The Abdus Salam International Centre for Theoretical Physics (ICTP), Trieste, Italy

**Keywords:** Biological physics, Computational biology and bioinformatics, Computational models, Coevolution, Evolutionary genetics

## Abstract

We introduce a simple model that describes the average occurrence of point variations in a generic protein sequence. This model is based on the idea that mutations are more likely to be fixed at sites in contact with others that have mutated in the recent past. Therefore, we extend the usual assumptions made in protein coevolution by introducing a time dumping on the effect of a substitution on its surrounding and makes correlated substitutions happen in avalanches localized in space and time. The model correctly predicts the average correlation of substitutions as a function of their distance along the sequence. At the same time, it predicts an among-site distribution of the number of substitutions per site highly compatible with a negative binomial, consistently with experimental data. The promising outcomes achieved with this model encourage the application of the same ideas in the field of pairwise and multiple sequence alignment.

## Introduction

It is commonly recognized that the process of evolution of the DNA sequences coding for proteins tends to conserve protein structure and function more than their sequence. In particular, the coevolution of residues inside a protein sequence entangles substitutions between contacting sites. For example, after a mildly destabilizing mutation at a site, the residues in contact with it may more easily fix mutations in order to adapt to the new situation and re-establish the original structural and functional balance. In the last twenty years, many investigations on coevolving residues have been performed showing that one can infer structural information from residue covariation in large multiple sequence alignments^1–7^. At the same time, models of protein evolution that incorporate structural information have been shown to better approximate the real evolutionary process^8,9^. These findings suggest an important contribution of structural coevolution in the process of sequence evolution and ensures that multiple mutations can affect the fitness of a protein sequence in a non linear way, phenomenon known as epistasis^10–13^. Moreover, it has been observed that the rate at which each site fixes random mutations varies both among sites^14–17^ and in time^18–20^ making the process of sequence evolution more complex than a simple set of identical and independent Markov processes on the protein sites^21^. In genetics, this feature is well known. The evolutionary time scales are usually defined according to a “molecular clock” that was originally modeled by taking sequence evolution as a set of independent and identically distributed Poisson processes^22,23^. In the last 30 years, several models have tried both to explain the overdispersion of this model^24–26^ and to propose more reliable ways to estimate evolutionary times^27^ both within neutral theory of evolution and invoking natural selection. Recently, it was shown that defining the evolutionary time scales according to the molecular clock is intrinsically biased, especially for proteins belonging to complex organisms, in which most of the sequence codes for complex structural motifs where random mutations are unlikely, and only highly correlated changes are possible^28^. Violations from the simple framework predicted by molecular clock and neutral theory can be quantified, for example, by estimating the so-called *overlap ratio*, namely the number of sites mutated more than once divided by the number of sites where at least a mutation took place^28^.

In the context of substitution rate variability, Fitch and Markowitz^18^ theorized that only a limited number of protein sites at a time can fix mutations, and when this happens other residues coupled with them will gain mutational freedom. This phenomenon would produce groups of *COncomitantly VARIable codONS* (for simplicity *covarions*) which vary over time in a correlated way. Unfortunately, despite this initial qualitative intuition, most of the quantitative implementations of the covarion model^29,30^ had to sacrifice the inclusion of any spatial pattern of covariation for the sake of computability.

In the line of thought introduced by the covarion model we here propose a simple model to describe the time evolution of the substitution rates of protein sites involved in structural and functional stability by explicitly including spatial and temporal interdependence. The core idea is that the probability of a mutation to be fixed at a site is enhanced if this site is in spatial proximity with other recently mutated sites, mimicking the mechanism of compensatory mutations. At variance with standard coevolutionary models, we assume that this mechanism acts only for a finite time: if compensatory mutations do not take place in a few generations the substitution rates of the neighboring sites go back to their baseline value as if the initial substitution was effectively forgotten. This, as we will show, allows reproducing several non trivial features observed in protein sequence evolution by a simple model.

Our model accurately reproduces the experimental patterns of along-chain conditional probability of substitutions in alignments in the sequence identity range 60–90%. Moreover, the number of substitutions per site produced by our model is well described by a negative binomial distribution, as generally found in phylogenetic analysis. The shape parameter of this negative binomial distribution falls in a realistic range and increases for growing evolutionary time in qualitative accordance with experimental data. This indicates that the model reproduces, at least qualitatively, a realistic distribution of substitution rates.

Our model predicts that substitutions take place in *avalanches* localized not only in three-dimensional space, as commonly predicted by coevolution, but also in time.

The simplicity of this model lies in an implementation which neglects the specific sequence, focusing only on contact maps and on the set of times of the last mutation of each site. Our results foster the hypothesis that the variability of substitution rates, both along-chain and in time, is strongly connected with coevolution and that a mutation triggers indeed the acceptance of other mutations in nearby sites only for a limited time. The minimal model described here, even if simple, may provide a handy implementation of combined space and time variation of substitution rates that might be included in more complex models. For example, it may be combined with a model of codon or amino acid substitutions^31–34^.

## Results

### Model

We developed a minimal dynamic model to describe the substitution rates in protein sequence evolution. Here mutations in the neighborhood of recently mutated sites have increased chances to be fixed by natural selection. For simplicity we model only substitutions, i.e. mutations fixed during the evolutionary process, and neglect those mutations which are destined to vanish. We will then indifferently label the time of appearance of a substitution as its time of mutation or time of substitution. This approximation becomes acceptable when dealing, as in our case, with time scales longer than the interval between the appearance and fixation of a mutation. This model accounts for realistic contact probabilities between the protein sites by means of model contact maps extracted from PDB structures^35^ hereafter denoted by ***C***, with *C*_*i*,*k*_ = 1 if sites *i* and *k* are in contact and *C*_*i*,*k*_ = 0 otherwise (see Materials and Methods for more details). Under the working hypothesis that a mutation at any given residue increases the mutation rate of all its spatially close neighbors, we model the substitution rate of site *i* at time *t* by

1 $${r}^{i}(t)={r}_{0}+J\sum _{k}\,{C}_{i,k}\,\exp [\,-\,(t-{t}_{k})]$$

where the sum runs over all protein sites and *t*_*k*_ corresponds to the time of the last mutation at site *k*. All times are measured in units of an implicit memory time and are thus dimensionless. There are two different terms involved in the right-hand side of Eq. (1). The first term consists in a constant rate *r*_0_ describing the mutational background, which for simplicity we first assume to be uniform. The second term accounts for the increase in the rate determined by mutations in one of the contacting residues. The role of the memory kernel exp[−(*t* − *t*_*k*_)] is to progressively reduce the impact of a substitution on the rest of the chain as time passes: if no other substitution appears in the neighborhood, after a sufficient amount of time, the substitution rates of that zone recovers their unperturbed value *r*_0_. From a biological point of view, this mimics the case in which a mutation keeps being transmitted from generation to generation without the early emergence of any compensatory mutation. In other words, this process mimics the occurrence of neutral mutations, i.e. mutations that are likely to have no significant detrimental effect on the protein structure and function. The inclusion of a memory kernel in Eq. 1 is the key novelty introduced in our model.

With this model, we perform a set of simulations in each of which we simulate the temporal evolution of two sequences which split at time zero from a common ancestor and are characterized by an empirical contact map sampled from real PDB structures (see Materials and Methods). The two sequences at time zero are identical and characterized by the same mutational history. With time passing, we keep track of the number of mutated sites and of the number of substitutions per site in the two branches. We then group these simulations by fraction of mutated sites and perform quantitative analysis separately for each sequence identity range *s* by averaging on different realizations of contact maps to compare them with data from real sequences.

We show in the supplementary material that the results depend very little from the value of *r*_0_ in the limit of small *r*_0_ (Fig. S2), while major differences are due to parameter *J*. The assumption of a unique *r*_0_ for all sites is here made for simplicity and will be relaxed in the section  Two-class model, to mimic the variability of the likelihood of neutral mutations along the protein sequence observed in multiple sequence alignment.

Notice that, since we do not include any information on the precise sequence of amino acids, each sequence *S* in our model is only characterized by a length *L* and by the times of latest mutation for each site {*t*_*k*_}_*k* = 1, …, *L*_. For the same reason, our estimate of the sequence identity after a given set of substitutions is only a lower bound, because it does not take into account the possibility that subsequent substitutions at the same site may restore the initial amino acid.

### Along-chain conditional probability of substitutions

We prepared several sets of ungapped local alignments of at least 80 residues starting from the UniRef database^36^, one per chosen sequence identity range (four bins respectively at 60–62%, 70–72%, 80–82% and 90–92%). We always use pairwise alignments in which one of the proteins is human, and the analysis is performed only for proteins with relatively high sequence identity with the human reference. This automatically excludes the vast majority of proteins belonging to simple organisms, such as yeast, where evolution can take place by different mechanisms^28^. For each sequence identity bin we collected at most one alignment per cluster per window of sequence identity to avoid uneven sampling (see precise procedure description in sec. Materials and Methods). Each alignment was translated to a binary sequence, with 0 corresponding to two identical paired amino acids (a persistence) and 1 to different paired residues (a substitution).

**Figure 1:**
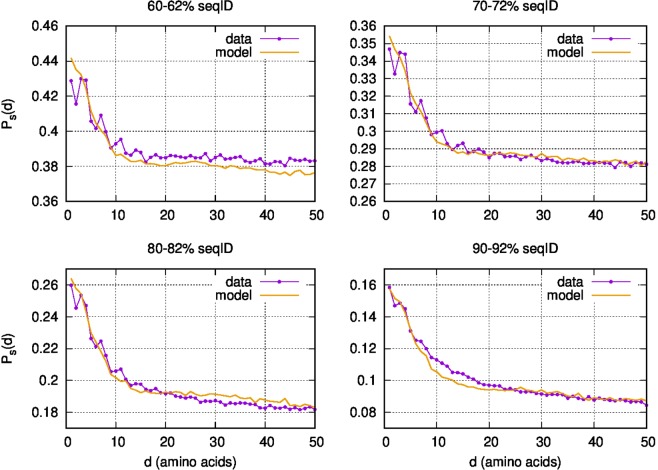
Conditional probability *P*_*s*_(*d*) of observing a mutation *d* sites away from another mutation at the sequence identities *s* respectively 60–62%, 70–72%, 80–82% and 90–92%. Model (*J* = 0.02 and *r*_0_ = 0.0004) in orange and data in purple.

For each analyzed sequence identity range *s*, we first investigate the along-chain conditional probability of finding a substitution *d* sites away from another one, ${P}_{s}^{data}(d)$, in the real pairwise alignment sets just described (purple curves in Fig. 1). This quantity exhibits a strong correlation decreasing with the distance. In the long-distance regime the conditional probability of observing two substitutions is, as expected, approximately equal to the single-point substitution probability.

Especially in the curves at lower sequence identity, the experimental pattern is perturbed by a short-range periodic modulation which is made evident by peaks in the conditional probability at distances of 3–4,7,10–11 and 15 amino acids. This modulation almost completely disappears after filtering out the sequences that JPred4^37^ predicts to have a fraction of *α*-helical residues larger than 38% (see Fig. S1 in SI). This is a strong hint that the spatial correlation is related with the structural contacts between residues.

We then compare ${P}_{s}^{data}(d)$ with the corresponding predictions of our models. We have here jointly optimized *r*_0_ and *J* on the four analyzed sequence identity ranges, obtaining *J* = 0.02 and *r*_0_ = 0.0004 (see Figs. S2 and S3 in SI respectively for different values of *r*_0_ and for separate optimizations at different sequence identities). The orange lines in Fig. 1 show the conditional probability of substitution for four different sequence identities as predicted by our model. It is evident that the model accurately reproduces the experimental probabilities in each investigated case. Sizable deviations from experimental data are visible only for 10 < *d* < 20 at 90% of sequence identity. This deviation may be due to our requirement of ungapped alignments (see Materials and Methods for more details), which at low sequence identity seems to select more structured regions with respect to higher sequence identity. With this in mind, it is then natural to expect, at different sequence identities, slightly different contact probabilities and, therefore, slightly different optimal values of *J*. In practice, the deviations of these values, which we attributed to the structural non uniformity in the dataset, are negligible. For simplicity we therefore consider both *r*_0_ and *J* as constant in the whole range of considered sequence identities.

### Distribution of the number of substitutions per site

In most algorithms for phylogenetic reconstruction based on likelihood maximization, the among-site rate variability is modeled by a Γ-distribution^14,15,38^ whose shape parameter *α* is estimated from the distribution of the number of substitutions per site^39,40^. Indeed, when dealing with a mixture of Poisson processes characterized by Γ-distributed rates, the number of substitutions per site is necessarily distributed according to a negative binomial whose shape parameter is the same *α*. The probability of observing *k* substitutions at a site is then:

2 $$p(k|\alpha ,\langle k\rangle )=\frac{\Gamma (\alpha +k)}{\Gamma (\alpha )\cdot k!}{(\frac{\langle k\rangle }{\langle k\rangle +\alpha })}^{k}\cdot {(\frac{\alpha }{\langle k\rangle +\alpha })}^{\alpha }$$

where 〈*k*〉 is the average number of substitutions per site computed from the data and *α* is the parameter to be estimated.

We here show that also our model produces distributions for *p*(*k*|*α*, 〈*k*〉) which are statistically compatible with a negative binomial, consistently with what inferred for real substitutions from phylogenetic trees^40^. These simulations are performed with the same values of *J* = 0.02 and *r*_0_ = 0.0004 discussed in the previous section.

**Figure 2:**
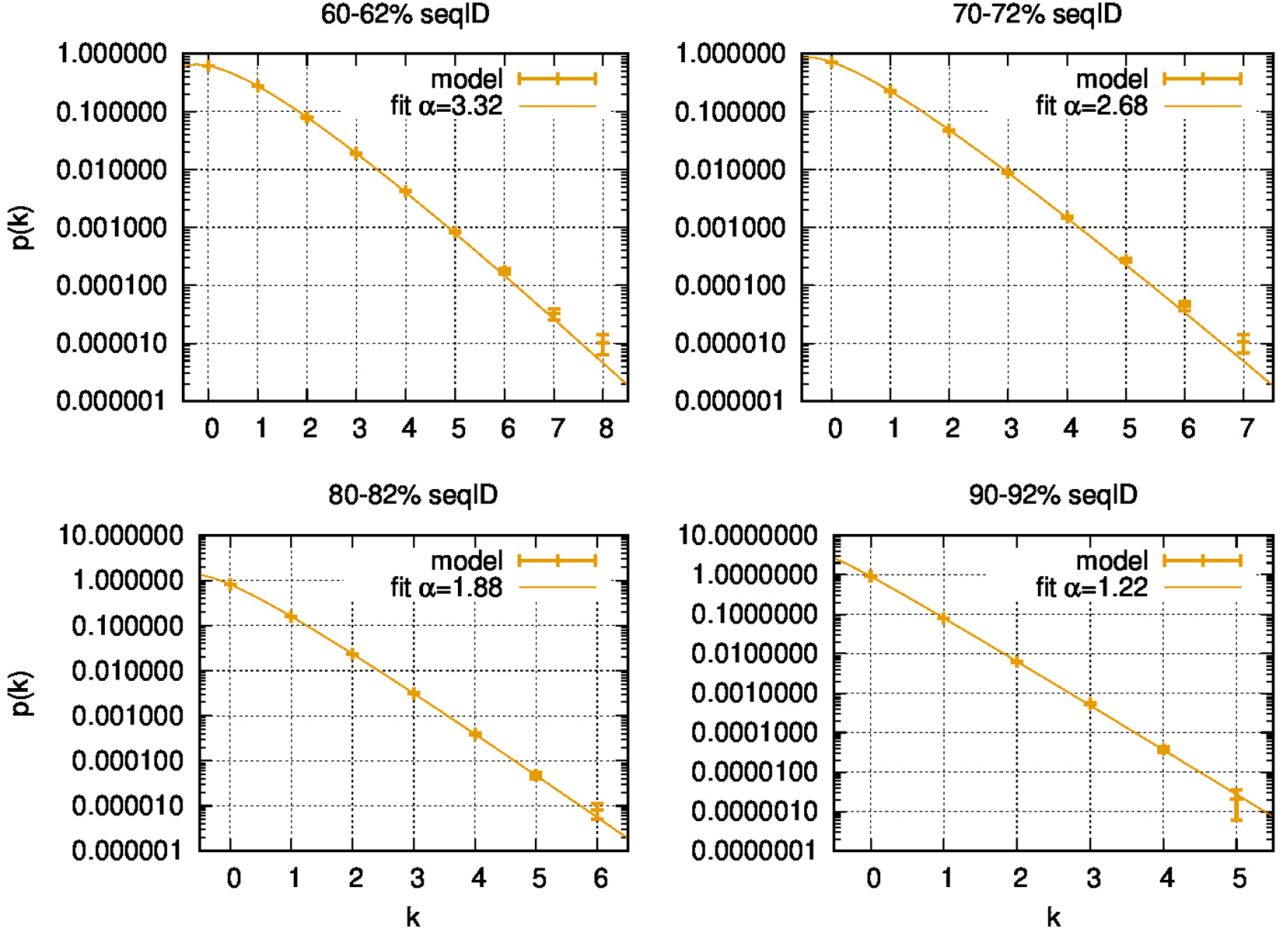
Weighted fit of the normalized histogram of the number of substitutions *k* per site to a negative binomial distribution at various sequence identities. It returns the best-fit value for *α* displayed in the key. The rms of residuals of these fits are respectively, from top-left to bottom-right: 1.9, 2.1, 0.33 and 1.3.

In Fig. 2 we show the normalized histogram of the number of substitutions per site *k* as simulated by our model at four different sequence identities. On top of each histogram we plot its weighted fit with a negative binomial (see Materials and Methods for the procedure). The negative binomial distribution well reproduces the statistics of the substitutions obtained by our model in all four cases.

**Figure 3:**
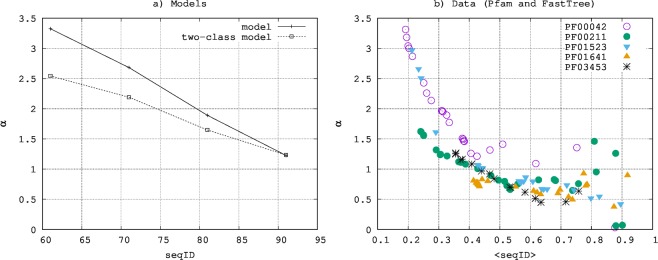
Panel (a): estimated *α* by the model described by Eq. 1 and by the two-class model as a function of the sequence identity. The value of *α* is estimated by fitting the number of substitutions per site to a negative binomial (Eq. 2). Panel (b): Estimate of *α* for five Pfam families obtained by FastTree-2^49^ on subtrees characterized by different average sequence identities (procedure described in Materials and Methods).

It is important to notice that in the proposed model each site has a rate that changes in time. This is very different to what happens in other models, for example those in which substitution rates are Γ-distributed but constant in time^14,15,40^. The shape parameter *α* is obtained by fitting to Eq. 2 the histogram of the number of substitutions per site in a given time interval. Therefore *α* characterizes the distribution of time-averaged substitution rates rather than instantaneous rates. As we reduce the sequence identity, the time interval over which we mediate grows, making the distribution of time-averaged rates diverge progressively from the distribution of instantaneous rates, becoming broader and broader. The solid line in panel a) of Fig. 3 quantifies this phenomenon by showing the value of *α* obtained by fitting to Eq. 2 the number of substitutions per site obtained by our model at different sequence identities. Similar results are found in real data, as also shown among others by^19,41^. In particular, to allow a visual qualitative comparison, the panel (b) in Fig. 3 shows the progressive growth in the estimate of *α* for decreasing average sequence identity when estimated from the multiple sequence alignments of five Pfam large families (see Materials and Methods for details). The values of *α* in panel (b) at growing sequence identity are estimated on smaller and smaller phylogenetic trees; thus these estimates become progressively less reliable, explaining the noisy cloud of points at sequence identity larger than 0.7. The increase of *α* predicted by our model is larger than the one observed in the examples of phylogenetic trees shown in figure. This indicates that the model introduced in this work reproduces this phenomenon only qualitatively. As we are going to see in the next section, introducing in the model a variability in the spontaneous substitution rate improves the qualitative agreement.

### Two-class model

The significant variation of *α* observed in our model, much bigger than what is found in real protein families, reasonably depends, among other effects, on our simplifying approximation of equal dynamics on all sites (neutral theory approximation). This assumption is known to be too simplistic: indeed in real proteins, flexible regions are typically more prone to fix mutations than structured regions. Moreover, part of the sequence codes for structural regions which are essential for the function of the proteins where the neutral theory cannot be considered a valid description^28^. In complex organisms, such as the ones considered in this study, the fraction of residues belonging to structural regions can be large. To verify the hypothesis that larger variations of *α* are due to the approximation of equal dynamics, we process the PDB structures used to build the contact matrices with the software STRIDE^42^, and classify each residue in one of two categories: structured or unstructured. Then, we make them evolve according to slightly different patterns. Both classes still obey Eq. 1, but with different parameters. For simplicity, we associate the same *J*^2*class*^ (different from before) to both classes, but we give them a different capability to fix new mutations (different *r*_0_). In particular, this capability will be very small for structured residues and we approximate it to zero. Therefore, structured residues will be characterised by ${r}_{0}^{S}=0$ and ${J}^{S}={J}^{2class}$, and unstructured residues by ${r}_{0}^{UNS}$ and ${J}^{UNS}={J}^{2class}$. We call this modified version of our model the two-class model. We optimise the two free parameters *J*^2*class*^ and ${r}_{0}^{UNS}$ by comparing the conditional probabilities ${P}_{s}^{two-class}(d)$ on the four sequence identity ranges as done in the previous sessions (Fig. S4 in SI), and we obtain the optimal values ${J}^{2class}=0.021$ and ${r}_{0}^{UNS}=0.01$.

When fitting to a negative binomial the histogram of the number of substitutions per site obtained with this two-class model, we find indeed smaller *α* variations: these values are shown in dashed line in panel (a) of Fig. 3 and, as supposed, present a much smaller increase at low sequence identity. Also in this case, the negative binomial distribution describes the distribution of the number of substitution per site fairly well (see Fig. S5 in SI). These results qualitatively demonstrate that allowing sites to differ in their average inclination to accept mutations leads to much more realistic estimates on *α* with respect to a model in which all sites are statistically identical. We also compute the overlap ratio^28^ in the two versions of the model in the analysed sequence identity bins and found that in all cases this value is bigger with the two-class model (see Table 1 in the Supplementary Material), showing once again that including information on structural variability makes the model more realistic.

## Discussion

Protein coevolution studies showed that substitutions exhibit a strong three dimensional spatial correlation due to compensatory mutations, which in turn is reflected on a correlation along the protein sequence. On the other hand, the substitution rates vary significantly among sites^14–17^ and in time^18–20^. Here we show how these two phenomena can be qualitatively described together by a simple model based on the idea that mutations may perturb the stability and functionality of a protein by introducing a frustration which dumps down in time. As a consequence, all sites that are in contact with a mutated one are themselves stimulated to accept mutations in order, for instance, to reduce frustration again. This idea has already emerged in the domain of protein sequence coevolution^1–7^: the novel ingredient that we introduce in this work is that this perturbation acts only for a finite time. In fact, if an isolated mutation has persisted for many generations, it is likely that it is neutral and has no effects on the fitness of the protein, so does not need compensation. We presented a model based on this idea, which revisits the covarion model proposed for the first time by^18^.

This model accurately reproduces the observable average along-chain correlation of substitutions in a large range of sequence identity. This suggests that the observed correlation may be due to structural contacts between residue pairs, as also confirmed by the peaks at the *α*-helical contact periodicity that vanish when the predicted *α*-helices are removed from the dataset (Fig. S1 in SI).

The two parameters of our simple models seem to fit the data fairly well at all the analyzed sequence identities. This suggests that sequence evolution can be approximated, in this framework, by a non-equilibrium stationary process, with features resembling those of the growth of a sand pile^43^.

Remarkably, the same model also reproduces a distribution of the number of substitutions per site largely consistent with a negative binomial, a distribution also empirically observed in phylogenetics. An overestimate of the shape parameter of this negative binomial distribution increasing with evolutionary time is visible both in real data and in our simulations and, at least in the model, is due to the time variation of the rates. We have also shown that the larger variability of the shape parameter in our model with respect to real data is likely to be due to the approximation of equal dynamics at all sites. This does not take into account the well known fact that in large regions of the proteins belonging to complex organisms random mutations are very unlikely. Indeed, we have shown that a trivial inclusion of differences in the mutation rates among sites (different *r*_0_ in our example) is already enough to bring the shape parameter closer to the one observed in real data.

**Figure 4:**
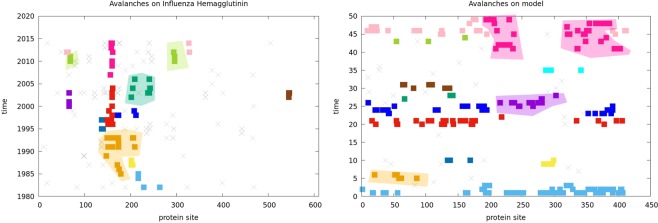
Panel (a): Substitution avalanches on Influenza Hemagglutinin from 1980 to 2015^44^. Panel (b): Avalanches of simulated substitutions (Eq. 1 with *J* = 0.02 and *r*_0_ = 0.0004) on an example structure (PDB 16pk, chain H). In both panels each cross or square represents one substitution which took place on the site corresponding to the *x* value and in the year corresponding to the *y* value. Gray crosses stand for either isolated substitutions or avalanches made by two substitutions. The squares label the remaining substitutions and are colored according to the avalanche to which they belong according to the procedure described in section  Avalanches detection and data from influenza hemagglutinin. The colored regions highlight some of the avalanches, and are only guides for the eye. Notice that the same avalanche can be split in two or more regions along the sequence, since a contact can be present even between sites which are not close along the sequence.

The model introduced in this work enhances the probability of substitutions taking place *at similar times and at contacting protein sites*. This process can be summarized by saying that our model produces *avalanches of substitutions* confined not only in space, as commonly expected by coevolution, but also in time. It is difficult to check if these avalanches exist in real cases, because to perform the check one needs to know the protein sequence in all intermediate evolutionary steps. This can however be done in a few cases where this strict condition is luckily verified. One of these cases is the evolution of the viral protein influenza Hemagglutinin: this protein, being important for vaccine conception and being then exposed to strong evolutionary pressure, varies quickly and has been largely studied and systematically sequenced in the last forty years^44^. In Fig. 4 panel (a) all substitutions found in the time evolution of yearly-based consensus sequences of Influenza Hemagglutinin between 1981 and 2015 are shown by squares or crosses placed in correspondence of its site (x axis) and its year of appearance (y axis). Gray crosses correspond to isolated or coupled substitutions, while the squares are colored in such a way that substitutions tagged by the same color are spatially and temporarily related. What we call “substitution avalanches” are these sets of spatially and temporarily related substitutions. Even if the exact partitioning in avalanches depicted in Fig. 4 is a consequence of the clustering approach (detailed in section  Avalanches detection and data from influenza hemagglutinin), the correlation of the substitution events is qualitatively visible even ignoring the color codes. Similar patterns can be observed in simulated evolution obtained by our model for a generic protein in the pdb (panel b). The case of Influenza Hemagglutinin is a clear case of positive selection, with variations happening on very fast time scales. It would be interesting to check if coupled spatial and temporal correlations are observed in more neutral frameworks, but we are not aware of any dataset giving similar information on time scales large enough to allow a quantitative comparisons with our model.

Our results seem to underline the importance of accounting for coevolution in the modeling of substitution rates. The minimal model described here may provide a handy implementation of combined space and time variation of substitution rates that might be included in a more complex framework and, for example, combined with a model of codon or amino acid substitutions. Moreover, in view of the observed pattern of along-chain correlation of substitutions, it would be interesting to account for an increased probability of nearby substitutions into the existing model and algorithms for protein sequence alignment, especially for pairwise alignments where no a priori information is available on the substitution rate or on the structural and functional constraints.

## Materials and Methods

### Experimental along-chain correlation of substitutions

We retrieved the protein sequences used to test our model from UniRef^36^, an arrangement of the UniProt database^45^ that clusters sequences above a certain sequence identity threshold. We downloaded the clusters at 50% of sequence identity with at least one human sequence. The alignments were downloaded on 23/07/2015 from UniRef at http://www.uniprot.org/help/uniref with query: [query:count: [2 TO*] length: [50 TO*] taxonomy:Homo sapiens (Human) [9606] AND identity:0.5].

From each of these clusters we detected the human sequence and aligned it with each of the others. We splitted the full range of 50–100% of sequence identity into windows of 2% of sequence identity each (50–52%, 52–54%, …, 98–100%) and then collected at most one alignment per cluster per window of sequence identity, to avoid weighting bigger clusters more. Here we used only the alignments at 60–62%, 70–72%, 80–82% and 90–92%. Sequences were aligned locally by the algorithm *water*^46^ in the *emboss* software package^47^ and only ungapped parts of at least 80 residues were considered.

Each alignment was translated to a binary sequence, with 0 corresponding to two identical paired amino acids (a persistence) and 1 to different paired residues (a substitution).

For each window of sequence identity *s*, we computed the overall conditional probability of observing a substitution *d* sites away from another substitution as the fraction

3 $${P}_{s}^{data}(d)=\frac{{N}_{s}^{1}(d)}{{N}_{s}^{0}(d)+{N}_{s}^{1}(d)}$$

where *N*_*s*_^1^(*d*) and *N*_*s*_^0^(*d*) are respectively the overall observed number of 1 and 0 found at a distance *d* from another substitution in the considered set of alignments. The first and last 5 residues of each alignments have been neglected to reduce boundary effects.

### Contact probability between protein sites

As model contact maps we downloaded the top500H database^35^, where the highly precise structures of 500 proteins are provided. For each structure, we computed the contact map by defining that two residues are in contact if their alpha-carbons are nearer than 10 Å.

We then ran STRIDE^42^ on these 500 structures to separate residues involved in secondary structured from those that are not. This separation has been used in the two-class model described in section Two-class model.

### Simulations and parameter optimization

Having observed that for the typical lengths in the test set of observed alignments the results depends on the lengths of the ungapped protein sequences, for each range of sequence identity we simulated many sequence lengths *L* whose distribution is compatible with the experimental one and obtained the desired quantities as averages over these different realizations (see Fig. S6 in SI for details).

The evolution of the rates on sequences of a given length *L* was simulated by randomly choosing one of the structures longer than *L*, selecting a portion of the protein of the desired length and evolving the protein on its whole length until when the selected portion reaches the desired sequence identity.

The evolution is performed by discretizing the time into small time steps *dt* and by computing the probability of substitution at each site *i* in such time intervals by:

$${p}_{i}(t)={r}_{i}(t)\cdot dt$$

Each site *i* mutates during that time step if a random number drawn from a uniform distribution in [0, 1] is smaller than *p*_*i*_. We verified that, with our choice of *dt*, two or more substitutions along the chain occurred at the same time step in less than 1% of the cases.

Before each simulation we ran an equilibration dynamics, allowing the initial times *t*_*k*_ (see Eq. 1) to reach values compatible with a stationary distribution. After the equilibration we evolved two sequences in parallel, both descending from the same original sequence (same initial *t*_*k*_ values). During each simulation we kept track of the number of mutated sites as well as of the number of substitutions per site (*k*_*i*_). We simulated the evolution of the two sequences until they reach the desired sequence identity. For each length we simulated many contact maps (being *L* part of a bigger protein among the 500 analyzed) and averaged on both lengths and contact maps. We assume that consecutive substitutions at the same site can not bring the site back to its initial amino acid type and that independent substitutions in the two branches do not give the same results. In other words, the number of mutated sites is simply the number of sites that mutated at least once in at least one of the two branches. A consequence of this fact is that what we call sequence identity is, more precisely, a lower bound of the sequence identity. However we expect this fact not to dramatically change any of the showed result.

We computed, according to our model, the conditional probability of finding a substitution *d* sites away from another one in sequences characterized by sequence identity *s* by

4 $${P}_{s}^{model}(d)=\frac{{N}_{s}^{1}(d)}{{N}_{s}^{0}(d)+{N}_{s}^{1}(d)}$$

Here ${N}_{s}^{1}(d)$ and ${N}_{s}^{0}(d)$ are respectively the total number of substitutions and persistences found at a distance *d* in the comparison of pairs of simulated sequences evolved with our model up to a sequence identity *s*. Also in this case the first and last 5 residues of each simulated sequences have been neglected to reduce boundary effects. For each range of sequence identity the simulations were stopped at the average sequence identity of the corresponding set of alignments. The optimization of parameters *J* and *r*_0_ have been accomplished by minimizing the root mean square displacement (RMSD) between the experimental ${P}_{s}^{data}(d)$ (Eq. 3) and ${P}_{s}^{model}$ in the four analyzed sequence identity ranges (60–62%, 70–72%, 80–82%, 90–92%). Only data corresponding to distances *d* along the chain shorter than 30 amino acids have been used during this optimization.

The weighted fit of Fig. 2 have been performed by gnuplot, with the errors computed according to the Poisson distribution. In the process of fitting, the average value of the distribution has been constrained to the experimental average value and so only the value of *α* was optimized.

### Estimation of *α* in pfam

We selected five Pfam families^48^ characterized by large multiple sequence alignments: PF00042, PF00211, PF01523, PF01641 and PF03453. For each of these families we downloaded the full multiple sequence alignment (download on March 7th 2016) and we built its phylogenetic tree (*T*_0_) by FastTree2^49^ with the inclusion of the Γ-correction. Given a tree, the level of each of its subtree is given by the number of edges between the root of the tree and the root of the subtree. Thus, starting from the root of this tree (*T*_0_), let us label *T*_1_ its 1-level subtree containing the largest number of leaves. From the common ancestor of subtree *T*_1_, we label *T*_2_ its most populated 1-level subtree, and so on until the leaves are reached. For each of these subtrees, we computed both the average sequence identity between the leaves, 〈*seqID*〉, and a new estimate of *α* recomputed from the sequences of that subtree only (again by FastTree2). These are the quantities shown in Fig. 3 panel (b). Subtrees of high level, associated to high average sequence identity, are characterized by a very small phylogenetic tree; thus, as evident from Fig. 3 panel (b), the associated value of *α* is difficult to determine and get progressively noisier and less reliable.

### Avalanches detection and data from influenza hemagglutinin

The sequences of Influenza Hemagglutinin used in Fig. 4 were downloaded on April 27^*th*^ 2016 from the NIAID Influenza Research Database^44^ by selecting protein data of virus type A and subtype H3N2 for the period 1981–2015 in Homo sapiens (complete segments only). Each sequence is characterized by a year, so its temporal evolution can be easily reconstructed. On average, the sequences of the same year are much more similar among themselves than to those of other years^50^. So, for each year, we computed a consensus sequence which has, at each position, the most common amino acid and we studied the evolution in time of these consensus sequences. From the PDB database^51^ we retrieved the entry 2WR0, containing one x-ray structure of the homotrimer of Influenza Hemagglutinin.

The sequences downloaded from the Influenza Research Database have all the same length and so they can be considered as a multiple sequence alignment. Using the program *PdbTool* (https://github.com/christophfeinauer/PdbTool.jl) we mapped each position in the sequence dataset to the corresponding residue on the PDB file.

From the PDB file we built a contact map between the protein residues by considering in contact two residues whose *α*-carbons are nearer than 8.5 Å. We mapped this contact map on the indexes of our MSA obtaining matrix *C*_*i*,*j*_, with *i* and *j* in the range 1 to 566 and *C*_*i*,*j*_ = 1 if the residues corresponding to sites *i* and *j* on the PDB are in contact and *C*_*i*,*j*_ = 0 otherwise. Not all the 566 residues in the MSA were mapped on a residue of the PDB, so we also added a contact between these sites not mapped on the the PDB file and their along-chain neighbors.

We compared the consensus sequences of consecutive years and kept track of mutations at each site *i* and time *t* by employing a mutation matrix *m*_*i*,*t*_, where *m*_*i*,*t*_ = 0 corresponds to a match between the amino acids at site *i* in the consensus sequences at times *t* and *t* − 1 and a *m*_*i*,*t*_ = 1 corresponds to a mismatch (implying that a mutation got fixed during that year). Thus, matrix *m* has size 566 (sites) × 33 (years).

We built a similar matrix *m* also for one of our simulations on protein 16 pk, chain H (PDB name) after discretizing their times to a number of bins comparable to the number of years available from influenza Hemagglutinin history.

From matrix *m*, we built an undirected graph whose nodes are labeled (*i*,*t*) by a site *i* and a year *t* and whose links connect nodes whose sites are in contact and whose associated times (year or bin) differ by no more than two units. An avalanche is then defined as a connected component of the graph.

## Supplementary information

Supplementary Figure 1
