# Supplementary material for: A common root for coevolution and substitution rate variability in protein sequence evolution
Source: Sci Rep. 2019 Dec 2;9:18032. doi: 10.1038/s41598-019-53958-w (PMC6888882; doi:10.1038/s41598-019-53958-w)
Supplement: Supplementary file 1 — Supplementary Figure 1 [file 41598_2019_53958_MOESM1_ESM.pdf]

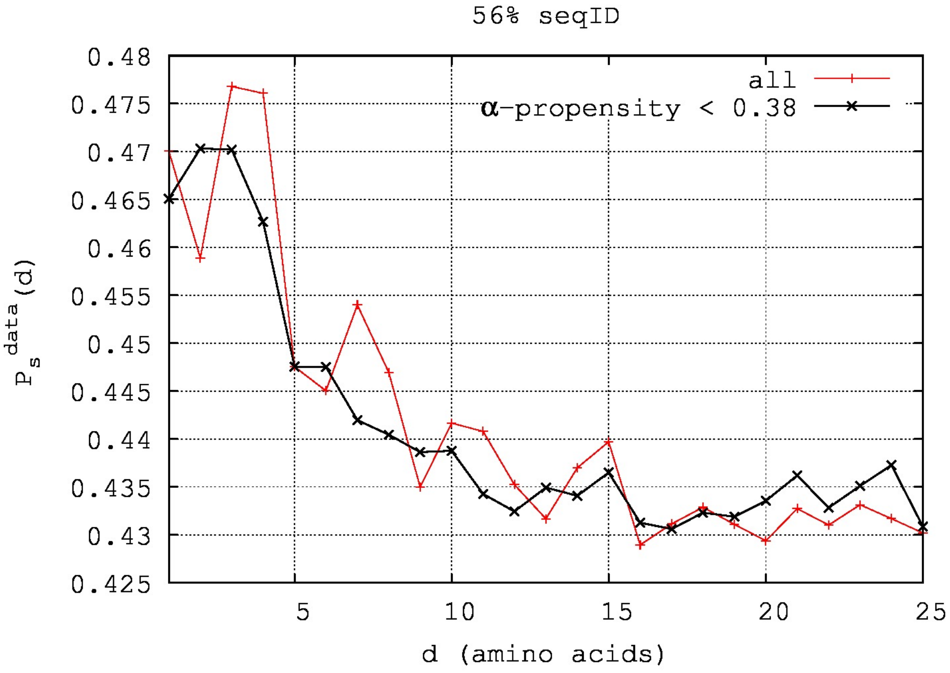

Figure S1. Effect of pruning alignments with high alpha helical content. Experimental conditional probability $P_s(d)$ of observing a substitution d sites away along the chain from another substitution at the sequence identity of 56% computed on all the available alignments (red) and on a subset characterized by α-helical propensity smaller than 0.38 according to the JPred4 predictor[37].

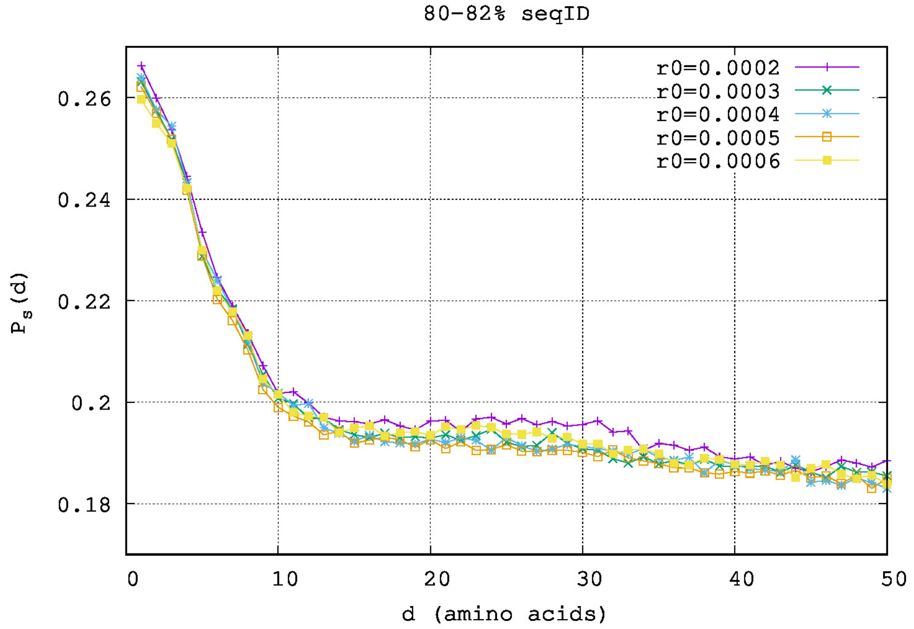

Figure S2. Different values of r_0. Conditional probability $P_s(d)$ of observing a substitution d sites away from another substitution obtained with our model in the sequence identity range 80-82% for different r_0 (see key). In each simulation J was fixed to 0.02.

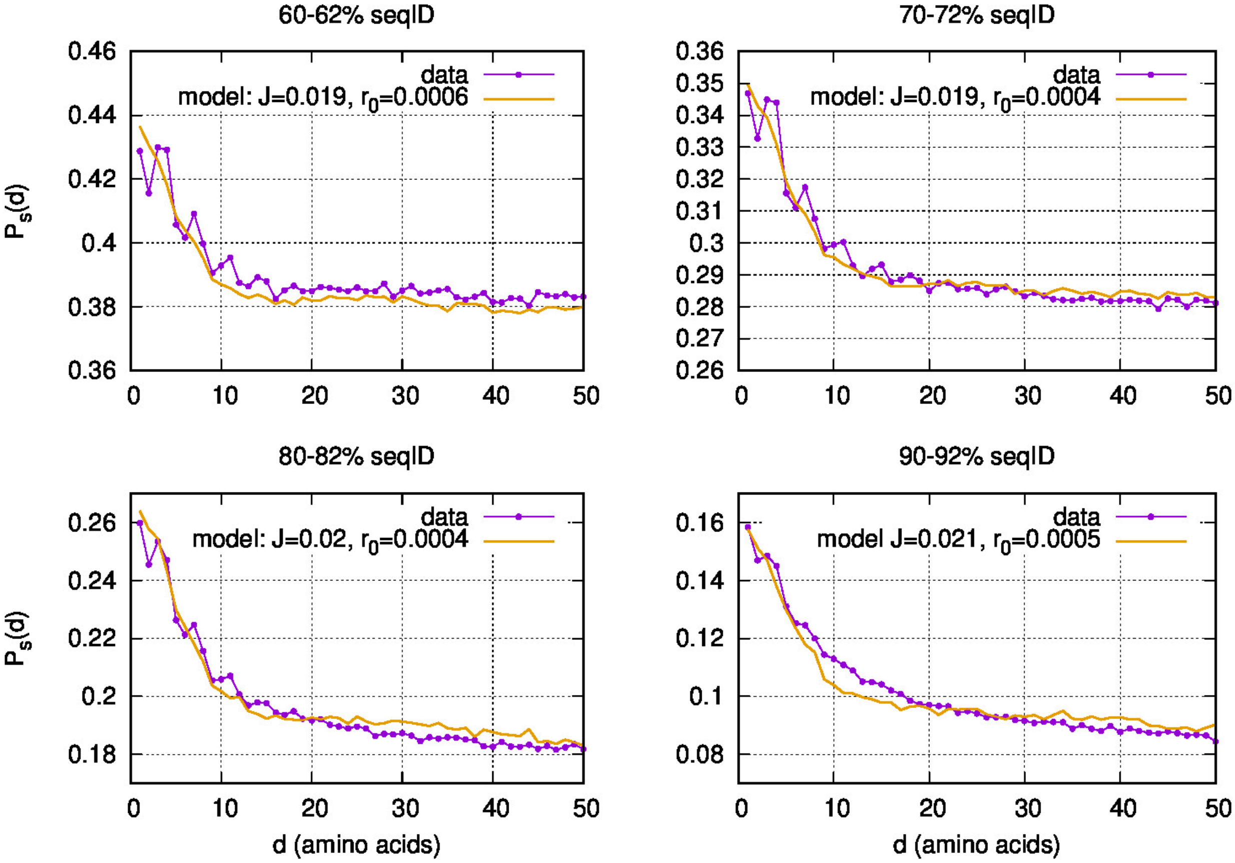

Figure S3. **Conditional probabilities with J and r_0 separately optimized.** Conditional probability $P_s(d)$ of observing a mutation d sites away from another mutation at the sequence identities s respectively 60-62%,70-72%, 80-82% and 90-92%. Model with J and r_0 separately optimized in orange and data in purple.

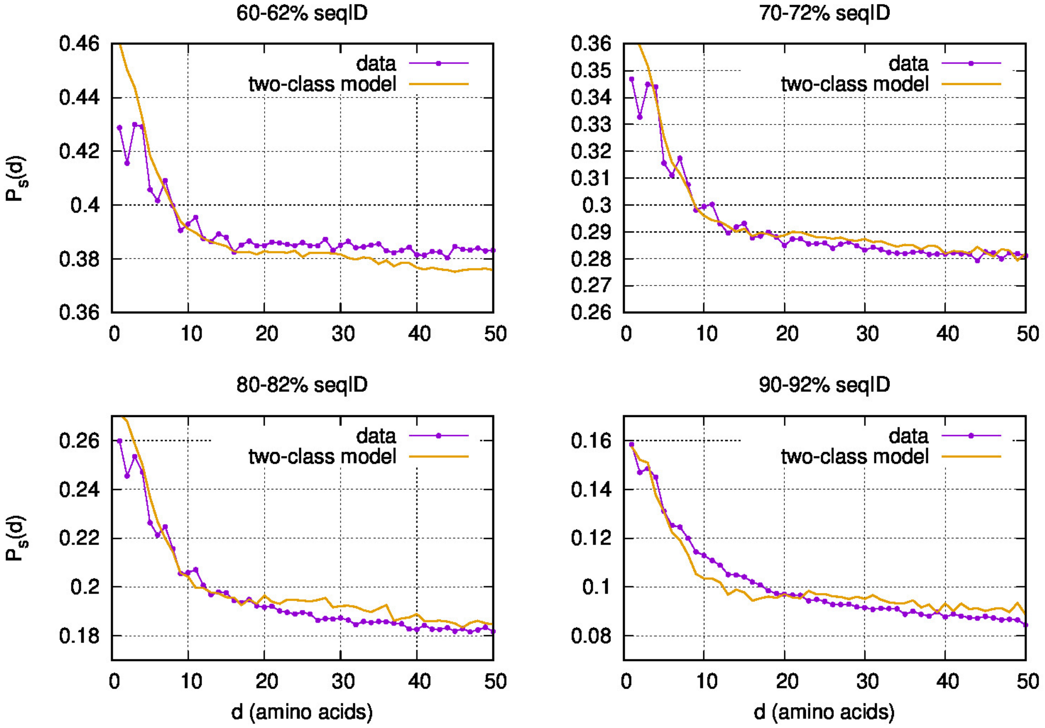

Figure S4. **Conditional probabilities with two-class model.** Conditional probability $P_s(d)$ of observing a mutation d sites from another mutation at various sequence identities s, respectively 60-62%,70-72%, 80-82% and 90-92%. Two-class model ($J^{2class} = 0.021$, $r_0^S = 0$ and $r_0^{UNS} = 0.01$) in orange and data in purple.

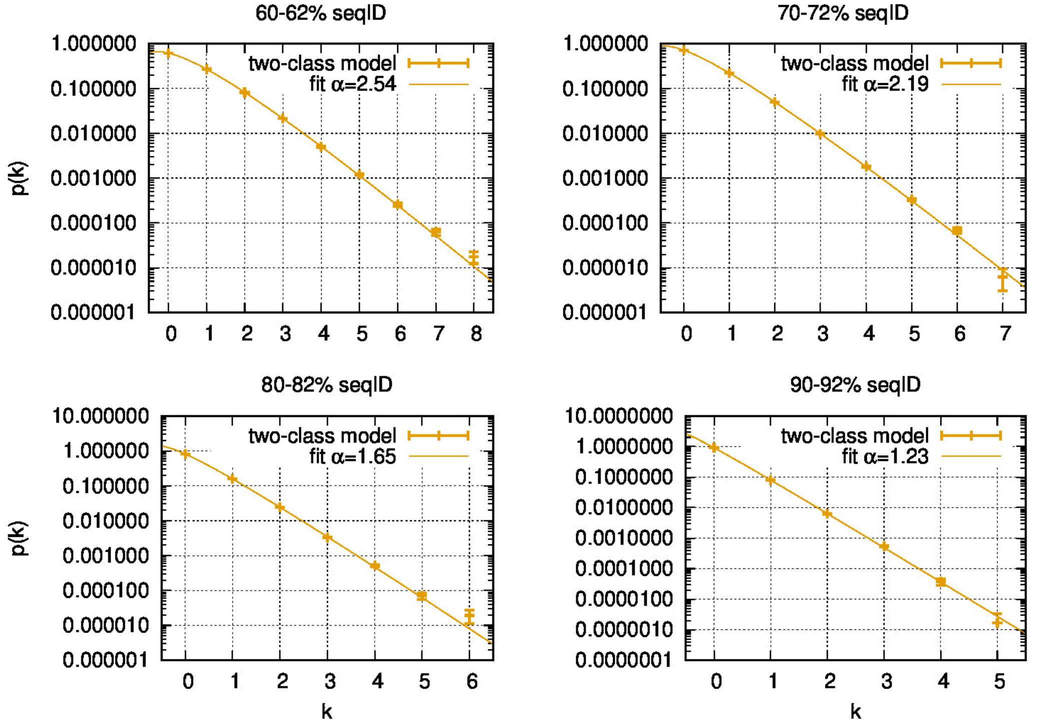

Figure S5. **Negative binomial fit for the two-class model.** Weighted fit of the normalized histogram of the number of substitutions per site k to a negative binomial distribution at various sequence identities for the two-class model ($J^{2class} = 0.021$, $r_0^S = 0$ and $r_0^{UNS} = 0.01$). The fit defines the value of α written in the key. The rms of residues of these fits are respectively, from top−left to bottom−right: 1.24,0.74,0.98 and 1.06.

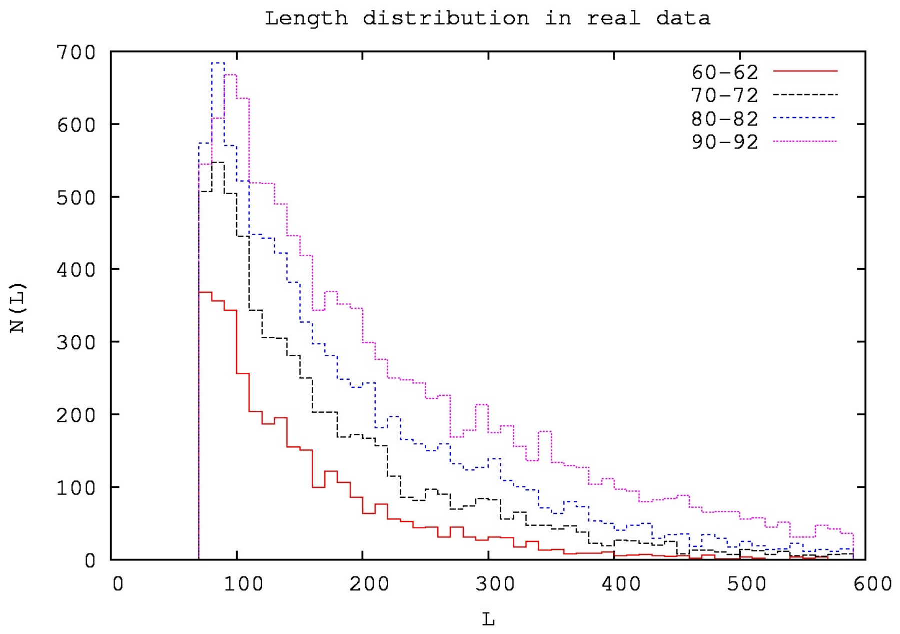

Figure S6. **Length distribution in real data.** Length (i.e. number of residues) distribution in the set of ungapped alignments from UniRef used to test the model, for the four considered sequence identity bins.

seqID	Overlap Ratio	Overlap Ratio two-class model
60-61%	0.271	0.288
70-71%	0.205	0.218
80-81%	0.146	0.153
90-91 %	0.0784	0.0785

Table 1. Overlap ratio for the simple model described in eq. 1 and for the two-class model. Here the overlap ratio is estimated as in[28]
